# Anti-Tumor Activity of Yuanhuacine by Regulating AMPK/mTOR Signaling Pathway and Actin Cytoskeleton Organization in Non-Small Cell Lung Cancer Cells

**DOI:** 10.1371/journal.pone.0144368

**Published:** 2015-12-11

**Authors:** Ji In Kang, Ji-Young Hong, Hye-Jung Lee, Song Yi Bae, Cholomi Jung, Hyen Joo Park, Sang Kook Lee

**Affiliations:** College of Pharmacy, Seoul National University, Seoul, 151–742, Republic of Korea; Suzhou University, CHINA

## Abstract

Yuanhuacine (YC), a daphnane diterpenoid from the flowers of *Daphne genkwa*, exhibited a potential growth inhibitory activity against human non-small cell lung cancer (NSCLC) cells. YC also suppressed the invasion and migration of lung cancer cells. However, the precise molecular mechanisms remain to be elucidated. In the present study, we report that YC significantly activated AMP-activated protein kinase (AMPK) signaling pathway and suppressed mTORC2-mediated downstream signaling pathway in H1993 human NSCLC cells. AMPK plays an important role in energy metabolism and cancer biology. Therefore, activators of AMPK signaling pathways can be applicable to the treatment of cancer. YC enhanced the expression of p-AMPKα. The co-treatment of YC and compound C (an AMPK inhibitor) or metformin (an AMPK activator) also confirmed that YC increases p-AMPKα. YC also suppressed the activation of the mammalian target of rapamycin (mTOR) expression, a downstream target of AMPK. Further study revealed that YC modulates mTORC2-associated downstream signaling pathways with a decreased expressions of p-Akt, p-protein kinase C alpha (PKCα), p-ras-related C3 botulinum toxin substrate 1 (Rac1) and filamentous actin (F-actin) that are known to activate cell growth and organize actin cytoskeleton. In addition, YC inhibited the tumor growth in H1993 cell-implanted xenograft nude mouse model. These data suggest the YC could be a potential candidate for cancer chemotherapeutic agents derived from natural products by regulating AMPK/mTORC2 signaling pathway and actin cytoskeleton organization.

## Introduction

Lung cancer is one of the most common diseases in the world and the leading cause of cancer-related death [1]. There are two main forms of the disease, small cell lung cancer (SCLC) and non-small cell lung cancer (NSCLC), where NSCLC is approximately 85% of all lung cancer cases. Along with surgery and radiotherapy, chemotherapy is one of the most common treatments for lung cancer therapy. However, chemotherapy is still not effective enough for patients with advanced NSCLC with a median survival rate of around one year [2, 3]. Therefore, the development of novel potent anticancer agents is still needed to treat the disease.

*Daphne genkwa* (Thymelaeaceae) is a well-known traditional medicinal plant distributed mainly in Korea and China, and its flower has been reported to exhibit abortifacient, anti-cancer, anti-tussive, diuretic, and expectorant activities [4]. Several compounds including daphnane-type diterpenes have been isolated from *D*. *genkwa* [5]. Daphane-type diterpenoids showed various biological effects including anti-cancer, transient receptor potential cation channel subfamily V member 1 (TRPV1) activating, anti-fertility, pesticide, neurotrophic, cholesterol-lowering, anti-hyperglycemic, irritant, tumor-promoting, and anti-human immunodeficiency virus (HIV) activities [6]. Yuanhuacine (YC) is a major component of daphnane-type diterpenoids isolated from the flower buds of *Daphne genkwa*. YC showed the anti-cancer activity in various cancer cell lines *in vitro* [7]. We also reported that YC exhibits the relatively selective growth inhibitory activity against human A549 lung cancer cells compared to the MRC-5 normal lung epithelial cells [8]. However, the underlying molecular mechanism of YC in human lung cancer cells has not been elucidated yet.

AMP-activated protein kinase (AMPK) is a ubiquitous serine/threonine protein kinase constituted of a catalytic α subunit and two regulator subunits (β and γ) [9], and is known to regulate cellular energy metabolism [10, 11]. Activation of AMPK is caused by cellular stress such as oxidative stress, hypoxia, and hypoglycemia, and it leads to increased ratio between cellular adenosine monophosphate (AMP) and adenosine triphosphate (ATP). AMPK also controls cell growth, proliferation and autophagy through the modulation of mammalian target of rapamycin (mTOR) activity, which is consistently deregulated in cancer cells [12]. There are two types of mTOR, mTORC1 and mTORC2 that are structurally and functionally different multi-protein complexes [13]. Generally, mTORC1 controls cell growth in response to nutrient availability and growth regulators. In contrast, mTORC2 is a key regulator of actin cytoskeleton that is correlated with cancer metastasis, and controls the phosphorylation of Akt at Ser-473 through the interaction between rapamycin-insensitive companion of mTOR (rictor) and mTOR [14, 15].

In the present study, the anti-tumor activity of YC and its underlying molecular mechanisms of action were investigated both in human H1993 lung cancer cells *in vitro* culture and in H1993-implanted xenograft nude mouse model *in vivo*.

## Materials and Methods

### Reagents

Dimethyl sulfoxide (DMSO), bicinchoninic acid (BCA), trichloroacetic acid (TCA), sulforhodamine B (SRB), and catalase were purchased from Sigma-Aldrich, Inc. (St. Louis, MO, USA), Roswell Park Memorial Institute (RPMI) 1640 medium, fetal bovine serum (FBS), trypsin-EDTA solution (1X), antibiotic-antimycotic solution (100X) and phosphate-buffered saline (PBS) (1X) were purchased from HyClone Laboratories, Inc. (South Logan, UT, USA). Anti-p-AMPKα, anti-AMPKα, anti-p-ACC, anti-ACC, anti-p-mTOR, anti-mTOR, anti-p-4EBP1, anti-4EBP1, anti-p-eIF4E, anti-eIF4E, anti-p-P70S6K1, anti-P70S6K1, anti-p-RPS6, anti-RPS6, anti-rictor, anti-p-Akt, anti-Akt, and anti-E-cadherin were purchased from Cell Signaling Technology (Danvers, MA, USA). Anti-PKC-α, anti-p-Rac1, anti-Rac1, anti-PCNA, anti-β-actin, and all secondary antibodies were purchased from Santa Cruz Biotechnology. (Santa Cruz, CA, USA). Anti-p-rictor was purchased from Millipore (Temecula, CA, USA). Anti-p-PKC-α, anti-F-actin, and anti-Ki-67 were purchased from Abcam (Cambridge, MA, USA). Gefitinib was purchased from Selleckchem (Houston, TX, USA) and rapamycin was purchased from Tocris Bioscience (Bristol, UK).

### Compound

**Fig 1:**
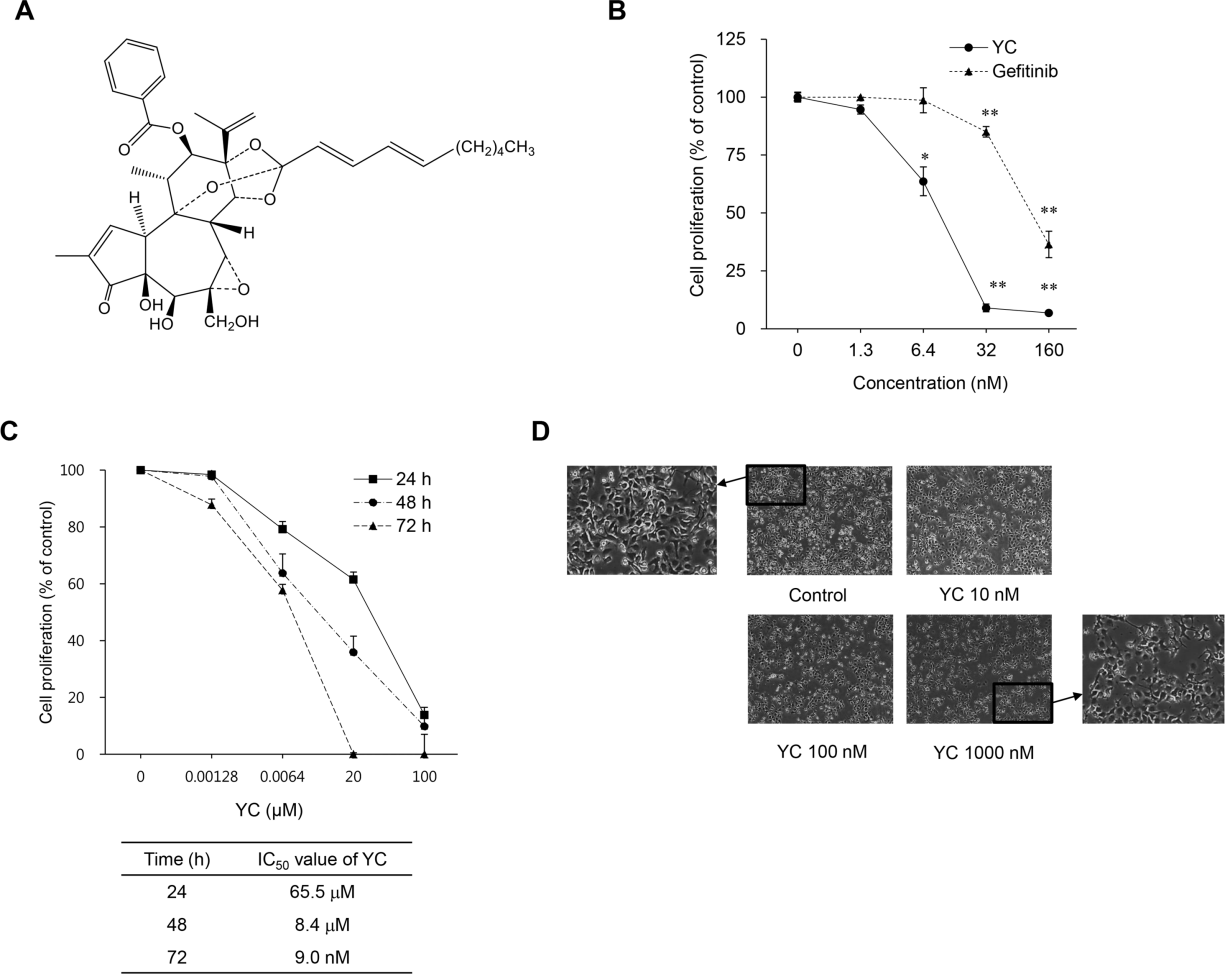
Growth-inhibitory effects of YC in H1993 NSCLC cells. (A) The chemical structure of YC. (B) H1993 cells were treated with the indicated concentrations of YC and gefitinib for 72 h. Cell proliferation was measured by SRB assay. (C) H1993 cells were treated with various concentration of YC for the indicated times, and cell proliferation was determined with the SRB assay. (D) Morphological changes mediated by the treatment of YC for 24 h were observed under the phase-contrast microscope.

Yuanhuacine (YC, Fig 1A) was isolated and identified from the flowers of *Daphne genkwa* as described previously [8]. The compound was dissolved in 100% dimethyl sulfoxide (DMSO) and diluted with medium for sample preparation.

### Cell Culture

The human NSCLC cell lines (H358, H460, Calu-1, H1299, A549, and H1993 cells) were purchased from the American Type Culture Collection (Manassas, VA, USA). The cells were cultured in RPMI1640 supplemented with 10% FBS and antibiotics-antimycotics (PSA; 100 units/mL penicillin G sodium, 100 μg/mL streptomycin, and 250 ng/mL amphothericin B) in a 37°C humidified incubator with 5% CO_2_.

### Cell Proliferation Assay

The effect of YC on cell proliferation was evaluated by SRB cellular protein-staining method. The cells were seeded in 96-well plates with various concentrations of samples and incubated at 37°C in a humidified incubator with 5% CO_2_. After 72 h of incubation, the cells were fixed with 10% TCA solution for 30 min and stained cellular proteins with 0.4% SRB in 1% acetic acid solution for 1 h. The stained cells were dissolved in 10 mM Tris buffer (pH 10.0). The effect of samples on cell viability was calculated as a percentage, relative to solvent-treated control. The IC_50_ values were calculated by non-linear regression analysis using the Table Curve 2D v5.01 software (Systat Software Inc., Richmond, CA, USA).

### Western Blot and Immunoprecipitation Analysis

For western blot analysis, the cells exposed to various concentrations of samples were lysed and protein concentrations were determined by BCA method. Total proteins (40 μg) in each cell lysate were subjected to resolution on various concentrations (6–15%) of sodium dodecyl sulfate-polyacrylamide gel electrophoresis (SDS–PAGE) and electro-transferred onto PVDF membranes. The membranes were incubated with blocking buffer (5% bovine serum albumin (BSA) in Tris-buffered saline and Tween 20 (TBST) for 1 h at room temperature and then further incubated with specific antibodies diluted in 2.5% BSA in TBST overnight at 4°C. After washing with TBST, the membranes were incubated with horseradish peroxidase (HRP)-conjugated secondary antibodies for 2 h at room temperature and visualized by HRP-chemiluminescent detection kit (Lab Frontier, Seoul, Korea) using LAS-4000 Imager (Fuji Film Corp., Japan).

For immunoprecipitation of the cultured medium, the cell supernatant was filtered through a 0.2 μm filter, and the protease and phosphatase inhibitors (Roche Applied Science, Penzberg, Germany) were than added. For immunoprecipitation of the cultured cells, the cells were lysed in IP lysis buffer (50 mM Tris-HCl pH 7.4, 150 mM NaCl, 1 mM EDTA, and 0.5% NP-40) containing protease and phosphatase inhibitors. The filtered cultured medium or equal amount of cell lysates was precleared for 30 min at 4°C with protein G Sepharose 4FastFlow (GE Healthcare, Little Chalfront, UK). After removal of beads (10 min, 12,000 rpm, 4°C), the supernatant was incubated with the indicated antibody overnight at 4°C. The immunocomplex was collected with beads at 4°C for 2 h. The beads were washed four times with IP lysis buffer, and the bound proteins were eluted with 2 x Laemmli sample buffer at 95°C for 10 min. The collected protein samples were subjected to western blot analysis.

#### Immunocytochemistry

The H1993 cells were sub-cultured on the cover slip coated with poly-L-lysine (Sigma-Aldrich, St. Louis, MO, USA) in a 12-well culture dish. The treated cells were washed three times with cold PBS, and were fixed in 2% para-formaldehyde for 15 min, followed by permeabilization with 0.5% Triton X-100 in PBS for 5 min. The cells were then washed with 0.1% Triton X-100 in PBS (PBS-T) and incubated in blocking solution (3% BSA and 1% normal goat serum in PBS-T) for 1 h. Primary antibodies were added to the blocking solution and the cells were incubated for 1 h at 37°C. After washing with PBS-T three times, the cells were incubated with appropriate secondary antibodies in blocking solution for 45 min at room temperature. The labeled cells were washed with PBS-T for six times and were mounted with mounting solution [100 mg/mL 1, 4-diazabicyclo [2.2.2] octane (DABCO) in 90% glycerol]. The slides were observed with a LSM710 laser scanning confocal microscope (Carl Zeiss, Germany).

### Cell Migration Assay

To assess the effect of YC on cell migration, a wound healing assay was performed as described previously [16]. Briefly, the H1993 cells were seeded in six-well plates and incubated for 48 h until they reached 80–90% confluences. A confluent monolayer of H1993 cells was artificially wounded with a micropipette tip, and the detached cells were washed with serum-free RPMI1640 and treated with test compounds in growing medium for 24 h. The cells were washed twice with PBS. Images of the wounds were photographed at 0 and 24 h under an inverted microscope (CKX41, Olympus, Tokyo, Japan).

### Cell Invasion Assay

The effect of YC on cell invasion was performed using the H1993 cells as described previously [17]. The cells were seeded into the top chambers of a 24-well Matrigel-coated polyethylene terephthalate membrane inserts with 8 μM pores (Millipore, Billerica, MA, USA). The plates were coated with 10 μL of type I chamber of the Matrigel-coated transwell insert. The medium of the lower chambers was also contained 0.1 mg/mL bovine serum albumin as a chemoattractant. The cells were incubated for 48 h at 37°C. The cells that had invaded the outer surface of the membrane were fixed with methanol, and stained with hematoxylin and eosin, and photographed (CKX41, Olympus, Tokyo, Japan).

### Analysis of Drug Combination

The cells (5 x 10^4^ cells/well) were plated in 96-well plates with various concentration of YC and rapamyacin or gefitinib. After 48 h of incubation, the cell proliferation was determined using the SRB assay. The combination effect was evaluated by calculating the combination index (CI) values as follows: CI = D_1_/(D_x_)_1_+D_2_/(D_x_)_2_, where D_1_ and D_2_ are the concentrations of the combined test compounds that achieve the expected effect, and (D_x_)_1_ and (D_x_)_2_ are the concentrations that achieve similar effects when the test compounds are used alone. In this study, 50% inhibition was taken as the effective level. The CI values were compared to the reference values reported by Chou [18].

### In Vivo Tumor Xenograft Study

Female nude mice [5 weeks old, BALB/c-nu (nu/nu)] were purchased from Central Laboratory Animal, Inc (Korea) and maintained in pathogen-free conditions. All animal experiments and care were conducted in a manner conforming to the Guidelines of the Animal Care and Use Committee of Ewha Womans University. The protocol was approved by the Committee on the Ethics of Animal Experiments of Ewha Womans University. H1993 cells were injected subcutaneously into the flasks of the mice (1 x 10^7^ cells in 200 μL of medium), and tumors were allowed to grow. When the tumor volume reached approximately 90 mm^3^, drug treatment was initiated. The mice were randomized into vehicle control and treatment groups of five animals per each. YC (0.5 or 1.0 mg/kg body weight) or gefitinib (10 mg/kg body weight) dissolved in a volume of 100 μl of solution (ethanol-Tween 80-H_2_O, 1:1:98) was administered orally once a day for 21 days. The control group was treated with an equal volume of vehicle of DMSO. Tumor volume was monitored for 21 days every 4~6 days using calipers, and tumor volume was estimated according to the following formula: tumor volume (mm^3^) = 3.14 x L x W x H / 6, where L is the length, W is the width, and H is the height.

### Immunohistochemistry of Tumors

Excised tumor tissues were fixed in 4% paraformaldehyde (PFA) and embedded in paraffin. Serial sections of the embedded specimens were deparaffinized, rehydrated, and subjected to antigen retrieval. The slides were incubated with the antibodies, which was detected using the LSAB+ System-HRP kit (Dako, Glostrup, Denmark) and counterstained with hematoxylin. Stained sections were observed and photographed under an inverted phase-contrast microscope.

#### Ex vivo biochemical analysis of tumors

A portion of the frozen tumors excised from the nude mice was thawed on ice and homogenized using a homogenizer in Complete Lysis Buffer (Active Motif, Carlsbad, CA, USA). Tumor lysates were subjected to protein concentration determination assays and aliquots were stored at -80°C.

### Statistical Analysis

Data were expressed as the mean ± standard deviation (S.D.). Statistical analyses were carried out using the Student’s *t-*test. Differences were considered statistically significant at *p<0.05, **p<0.01.

## Results

### Growth inhibitory effects of YC in cultured H1993 cells

Our previous studies have demonstrated that YC has a relatively selective growth inhibitory activity against human lung cancer cells compared to normal lung epithelial cells [8]. Therefore, in the present study, we extended to evaluate the anti-proliferative activity of YC in various NSCLC cell lines. YC exhibited a potential growth inhibitory activity in cultured human NSCLC cells (Table 1). In particular, the H1993 cell line was shown to be the most sensitive with the IC_50_ value of 9 nM, after 72 h incubation, and YC seemed to be more potent than gefitinib, an anticancer drug for treatment of NSCLC patients in clinic, under the same experimental condition (Fig 1B). When treated with various concentrations of YC for several time points (24, 48 and 72 h), YC inhibited the proliferation of H1993 cells in a concentration- and time-dependent manner (Fig 1C). Because the H1993 cell line was the most sensitive cell to YC, the mechanism action study for the anti-proliferative activity of YC was performed in the H1993 cells.

**Table 1:** Anti-proliferative activity of YC in various NSCLC cells, IC_50_ (μM).

Cell line	YC	Gefitinib
H358	16.5	8.0
H460	6.2	>200
Calu-1	4.1	53.0
H1299	4.0	4.5
A549	0.03	11.7
H1993	0.009	0.1

The morphological changes of the YC-treated cells were observed under the phase-contrast microscope after 24 h incubation. The cell numbers were decreased in a concentration-dependent manner, and the polygonal shape of control cells was changed into constricted and irregular dendrite-shape of cells (Fig 1D).

### Effects of YC on the AMPK signaling pathway

**Fig 2:**
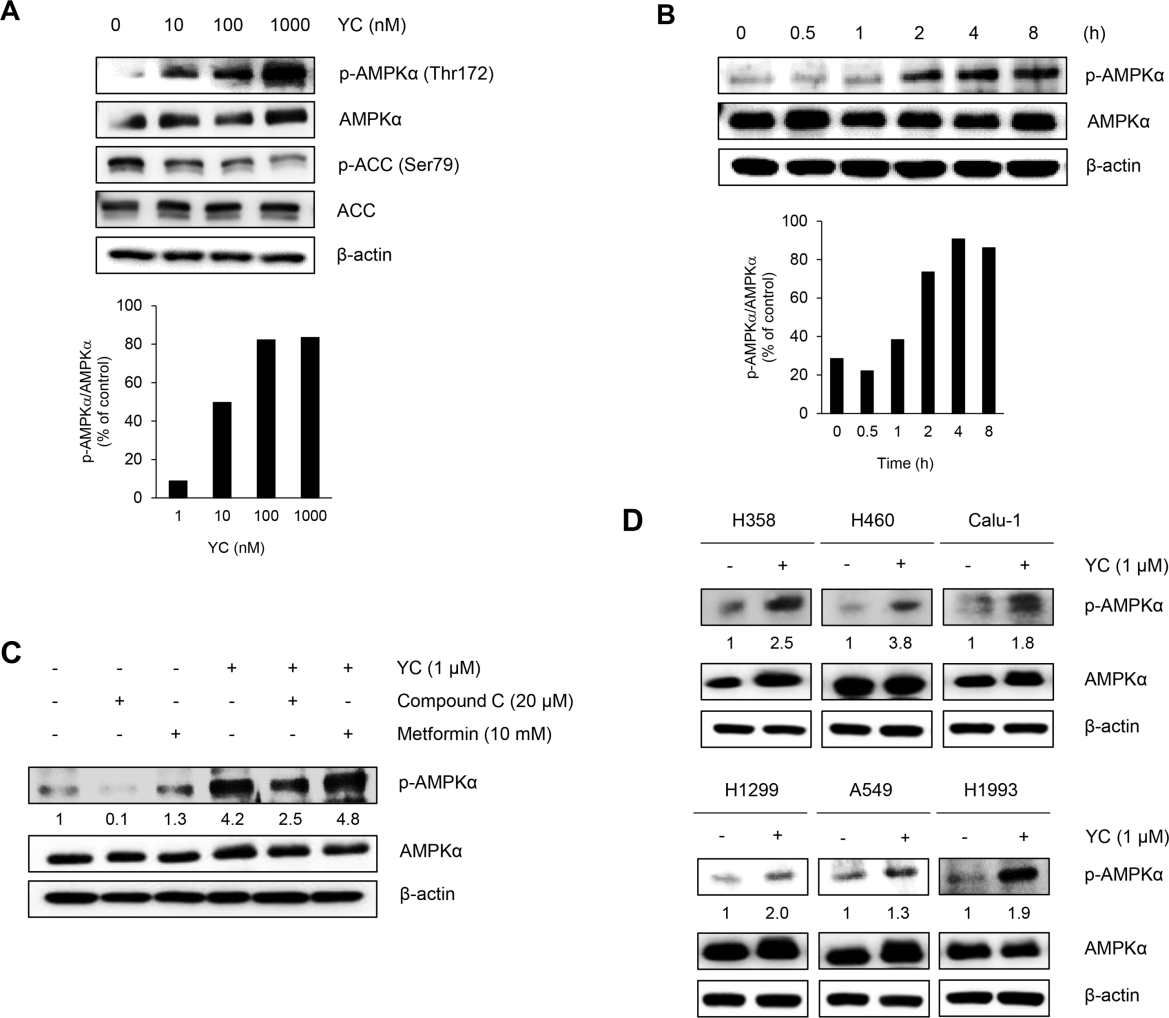
Effects of YC on the AMPK signaling pathway. (A) H1993 cells were treated for 24 h with the indicated concentrations of YC and the protein expressions were measured by Western blot analysis. (B) H1993 cells were treated for different short time points with 1 μM of YC and the protein expressions were measured by Western blot analysis. (C) H1993 cells were pretreated for 1 h with 20 μM of compound C, which is a well-known for AMPK inhibitor or 10 mM of metformin that is an AMPK activator. Then, 1 μM of YC was treated or co-treated for 2 h and the protein expressions were measured by Western blot analysis. (D) H358, H460, Calu-1, H1299, A549 and H1993 cells were treated for 2 h with 1 μM of YC and the protein expressions were measured by Western blot analysis.

To further elucidate the anti-proliferative mechanism of YC, the regulation of a cellular signal transduction pathway associated with cancer cell growth was analyzed using Western blot. AMPK is considered as a key molecule that controls cell growth, proliferation, and autophagy. After 24 h treatment with YC, the protein level of p-AMPK was significantly increased, and the level of p-acetyl-CoA carboxylase (ACC), a downstream target of AMPK, was subsequently decreased in a concentration-dependent manner (Fig 2A). Since AMPK activation is a very early event [19, 20], the activation of AMPK by YC was also monitored during a short period of time. The p-AMPK level was measured up to 8 h after treatment of YC (1 μM). The protein level of p-AMPK was significantly increased after 2 h incubation in a time-dependent manner in cultured YC-treated H1993 cells (Fig 2B). The activation of AMPK by YC was further confirmed with the pretreatment of compound C (a specific inhibitor of AMPK) or metformin (an AMPK activator). The H1993 cells were pretreated with compound C (20 μM) or metformin (10 mM) for 1 h and then co-treated with YC (1 μM) for an additional 2 h. Compound C suppressed the expression of activated AMPK (p-AMPK), but the co-treatment of YC recovered the AMPK activation. Similarly, YC also significantly enhanced the metformin-mediated activation of AMPK (Fig 2C). In addition, YC effectively activated the p-AMPK level in various NSCLC cell lines (Fig 2D). These findings suggest that AMPK can be a target protein in the anti-proliferative activity of YC in human NSCLC cells.

### Effects of YC on the mTOR signaling pathway

**Fig 3:**
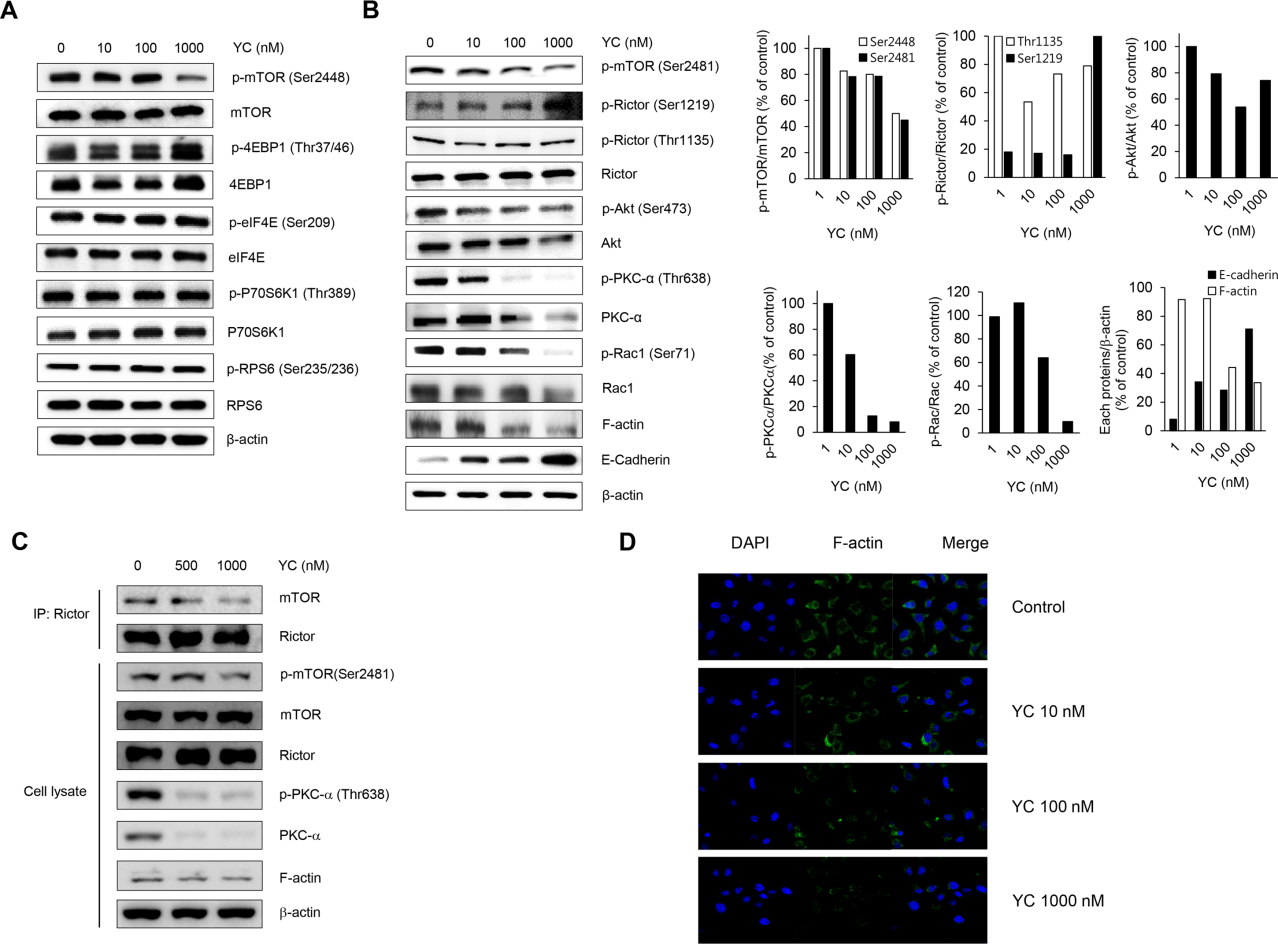
Effects of YC on the mTOR signaling pathway. (A) H1993 cells were treated for 24 h with indicated concentrations of YC and the expressions of mTORC1 signaling-related proteins were measured by Western blot analysis. (B) H1993 cells were treated for 24 h with indicated concentrations of YC and the expressions of mTORC2 signaling-related proteins were measured by Western blot analysis. (C) The interaction of mTOR and rictor have been analyzed by immunoprecipitation of rictor with the following immunoblots of the cell lysates (lower panel) and immunoprecipitates (upper panel) with the indicated antibodies. IP: immunoprecipitation. (D) H1993 cells were treated for 24 h with the indicated concentrations of YC and the F-actin expression was measured by immunocytochemistry.

It is well known that AMPK negatively regulates mTOR signaling pathway. Since AMPK was activated by YC in the H1993 cells, we further investigated whether YC also affects mTOR signaling pathway. We found that YC downregulated the activation of mTOR (p-mTOR (Ser2448)). Therefore, mTOR-associated downstream protein expressions were further determined. Since YC did not modulate the levels of mTORC1-related proteins such as 4E-binding protein 1 (4EBP1), eukaryotic translation initiation factor 4E (eIF4E), p70S6 kinase 1 (p70S6K1), and ribosomal protein S6 (RPS6) (Fig 3A), YC was further analyzed for effect on another mTOR complex, mTORC2, a modulator of actin cytoskeleton organization. As a result, YC suppressed mTOR autophosphorylation at Ser2481, a molecular biomarker for mTORC2 activity (Fig 3B). To investigate whether YC inhibited mTORC2 activity by affecting the association of mTOR with rictor, rictor was immunoprecipitated from YC-treated cells, and mTOR and rictor antibodies were detected by immunoblotting respectively. As shown in Fig 3C, YC disrupted the association of mTOR and rictor, and these events subsequently suppressed the activation of the mTORC2-associated downstream targets including Akt, protein kinase C alpha (PKC-α), ras-related C3 botulinum toxin substrate 1 (Rac1), and filamentous actin (F-actin) by YC treatment (Fig 3B). YC also activated the expression of a cell adhesion molecule E-cadherin, a negative regulator of F-actin in the H1993 cells (Fig 3B). The suppressive effect of YC on the expression of F-actin was also confirmed by immunocytochemical analysis (Fig 3D), suggesting that YC effectively inhibits the cytoskeleton organization of NSCLC cells. These findings are consistent with the observation of cell morphological changes with the treatment of YC in the H1993 cells.

### Effects of YC on invasion and migration

**Fig 4:**
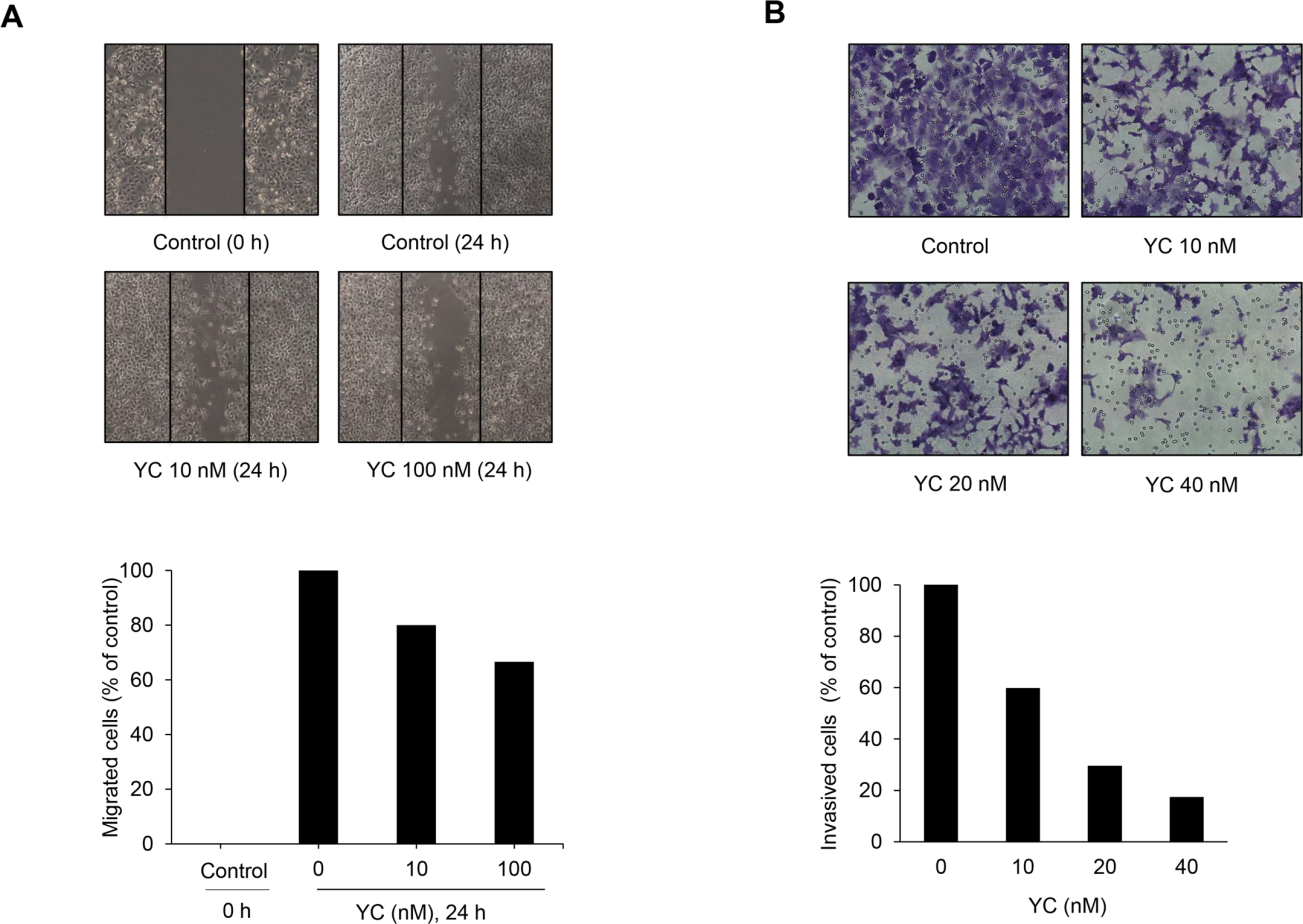
Inhibitory effects of YC on cell migration and invasion in H1993 cells. (A) H1993 cells were incubated in a 12-well plate for 24 h, wounded with a yellow tip, and treated with the indicated concentrations of YC. After incubation for 24 h, the cells were photographed using a phase-contrast microscope. (B) H1993 cells were treated for 48 h with the indicated concentrations of YC. The cell invasion assay was performed in a Matrigel-coated chamber system as described in Materials and Methods.

It is well known that the asymmetric accumulation of F-actin is associated with cell invasion and migration. YC suppressed the expression of F-actin in NSCLC cells. Therefore, we further evaluated the effect of YC on cancer cell invasion and migration. The treatment of YC for 24 h inhibited the migration of cancer cells in a wound healing assay in a concentration-dependent manner (Fig 4A). Additionally, Matrigel invasion assay was performed to study whether YC influences the invasiveness of malignant cancer cells. YC prevented cancer cells from moving into a Matrigel chamber in a concentration-dependent manner (Fig 4B). These data suggest that YC effectively inhibited the migration and invasion of cancer cells which is in part associated with the suppression of actin cytoskeleton organization via the downregulation of F-actin and upregulation of E-cadherin expression.

### Anti-proliferative effects with combination of cancer chemotherapeutic agents

**Fig 5:**
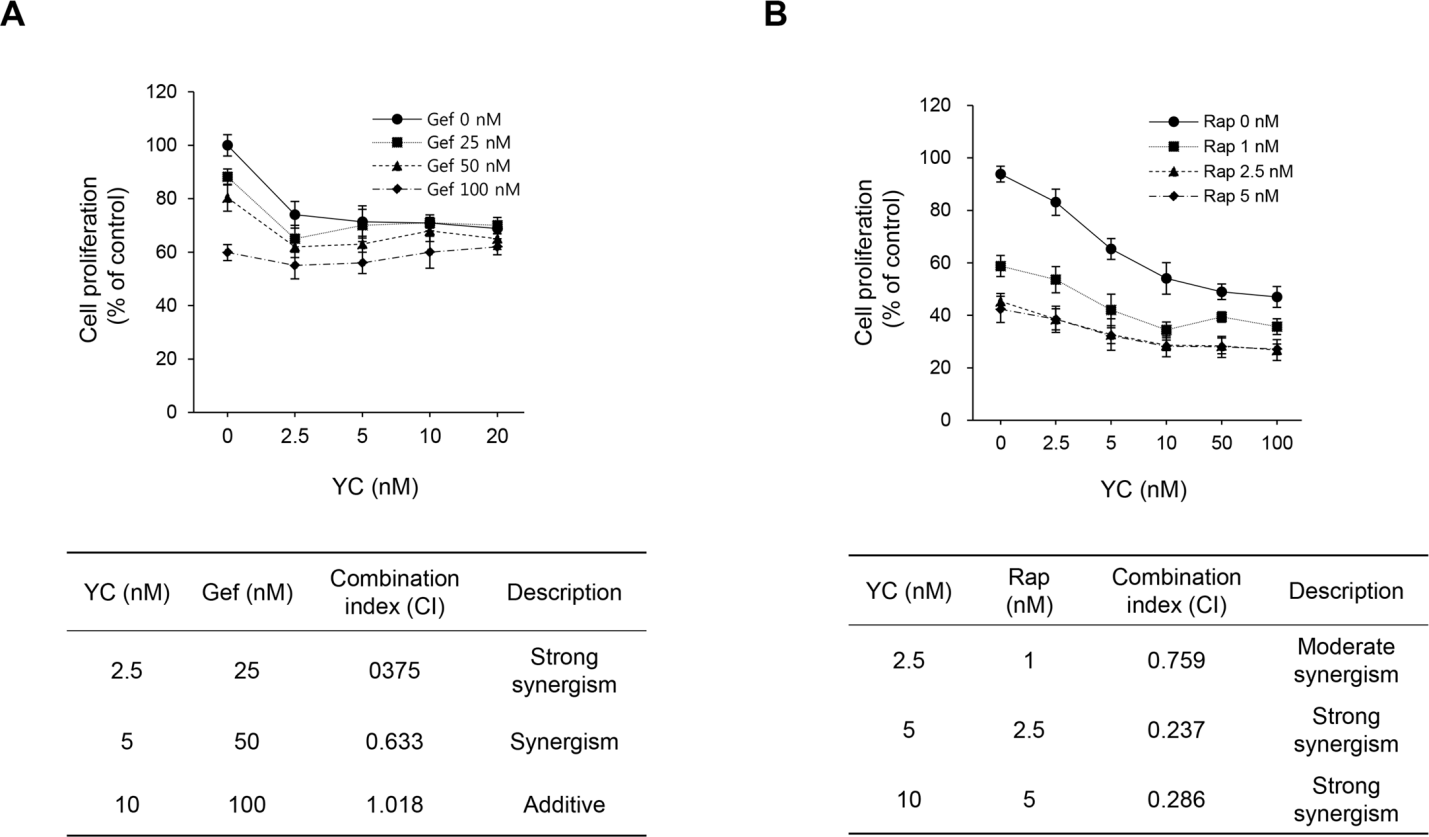
Anti-proliferative effect of YC in combination with gefitinib or rapamycin in H1993 cells. The cells were treated with YC alone or in combination with gefitinib or rapamycin for 48 h. The cell proliferation was measured using a SRB assay. (A) YC in combination with gefitinib (B) YC in combination with rapamycin.

We further examined the effect of YC in combination with gefitinib or rapamycin on H1993 cell proliferation. Several studies have reported the beneficial effects of combination of gefitinib, an EGFR inhibitor, with mTOR inhibitors in preclinical model. In this study, the combination of YC with gefitinib displayed the significant synergistic inhibitory effects on the H1993 cells (Fig 5A). In addition, the combination of rapamycin (an mTORC1 inhibitor) and YC effectively inhibited the cell proliferation of H1993 cells compared with rapamycin alone (Fig 5B). It is known that rapamycin blocks mTORC1 and does not inhibit mTORC2. mTORC1 inhibition without suppressing mTORC2 can activate PI3K/Akt and promote tumor survival. Therefore, this result suggests that the growth inhibition of YC combination with rapamycin could be due to the down-regulation of mTORC2 by YC.

### Inhibitory effect of tumor growth by YC in the H1993 cells implanted xenograft mouse model

**Fig 6:**
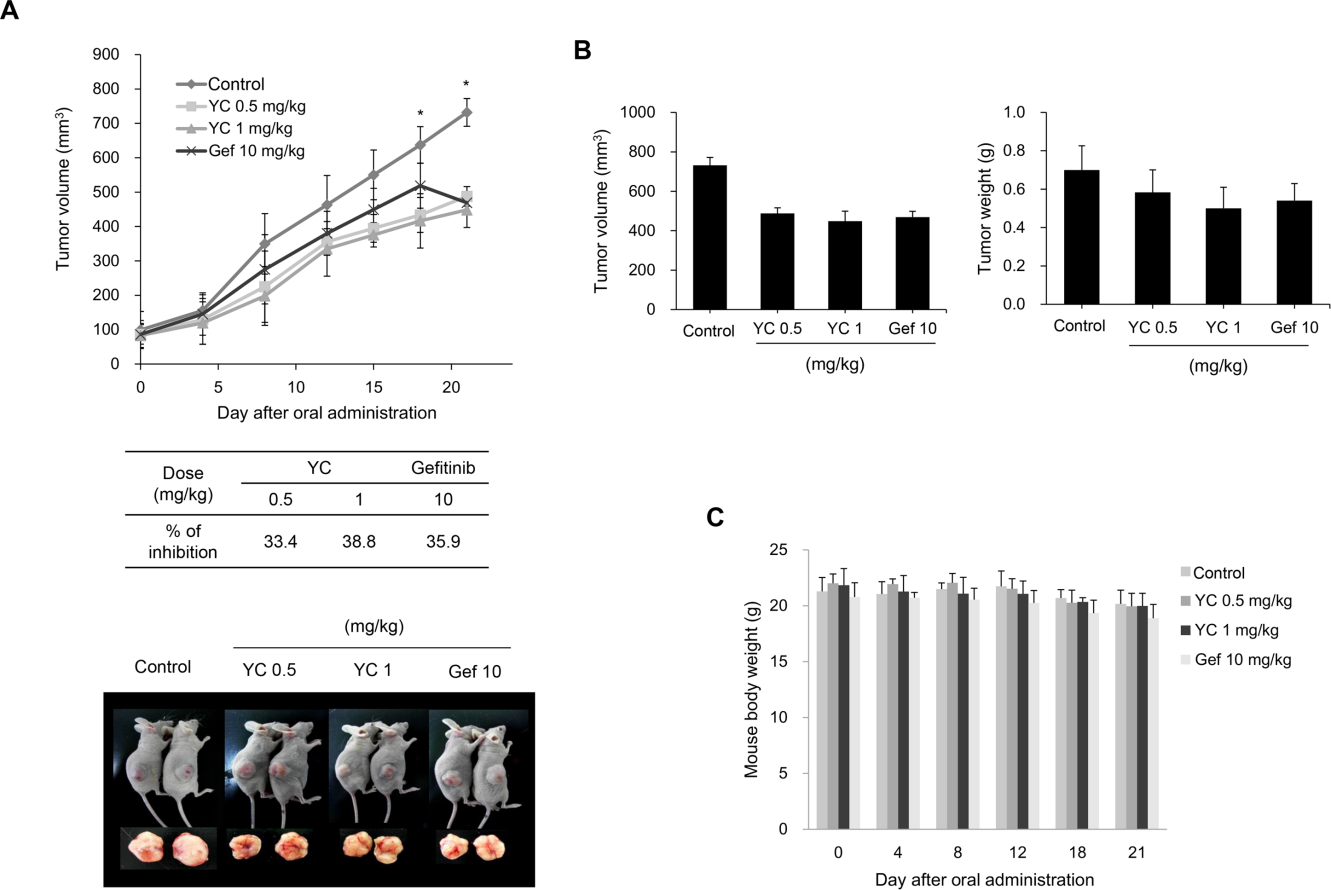
Inhibition of tumor growth by YC in H1993 xenograft model. (A) H1993 cells were injected s.c. into the flanks of nude mice and tumors, and were allowed to develop for 21 days. When they reached approximately 90 mm^3^, the indicated concentrations of YC were initiated. All study medications were given orally once daily for 21 days. Tumor sizes were monitored every 4~6 days. (B) The volume and weight of each tumor xenograft were measured at the termination of the experiment on day 21. (C) Animals in the control and treated groups were weighed every 4~6 days. The mean body weight of each group is presented.

The anti-tumor activity of YC was evaluated using an *in vivo* nude mouse xenograft model bearing H1993 cells. When tumor volume reached approximately 90 mm^3^ after implanted with the H1993 cells, YC (0.5 or 1 mg/kg of YC) or gefitinib (10 mg/kg) was orally administered to mice once a day for 21 days. The tumor volume in the vehicle-treated control group was approximately 750 mm^3^ in 30 days after the cells were subcutaneously implanted into the right flank region of each mouse. Compared to the vehicle-treated control groups, YC significantly inhibited the tumor growth by 33.4% and 38.8% at 0.5 mg/kg and 1.0 mg/kg, respectively, at the end of the experiments (Fig 6A). Tumor weights were also significantly inhibited by the treatment of YC (Fig 6B). Similar results were observed in the treatment of gefitinib (10 mg/kg). No body weight changes and overt toxicity were found in *in vivo* experiments with YC (Fig 6C).

**Fig 7:**
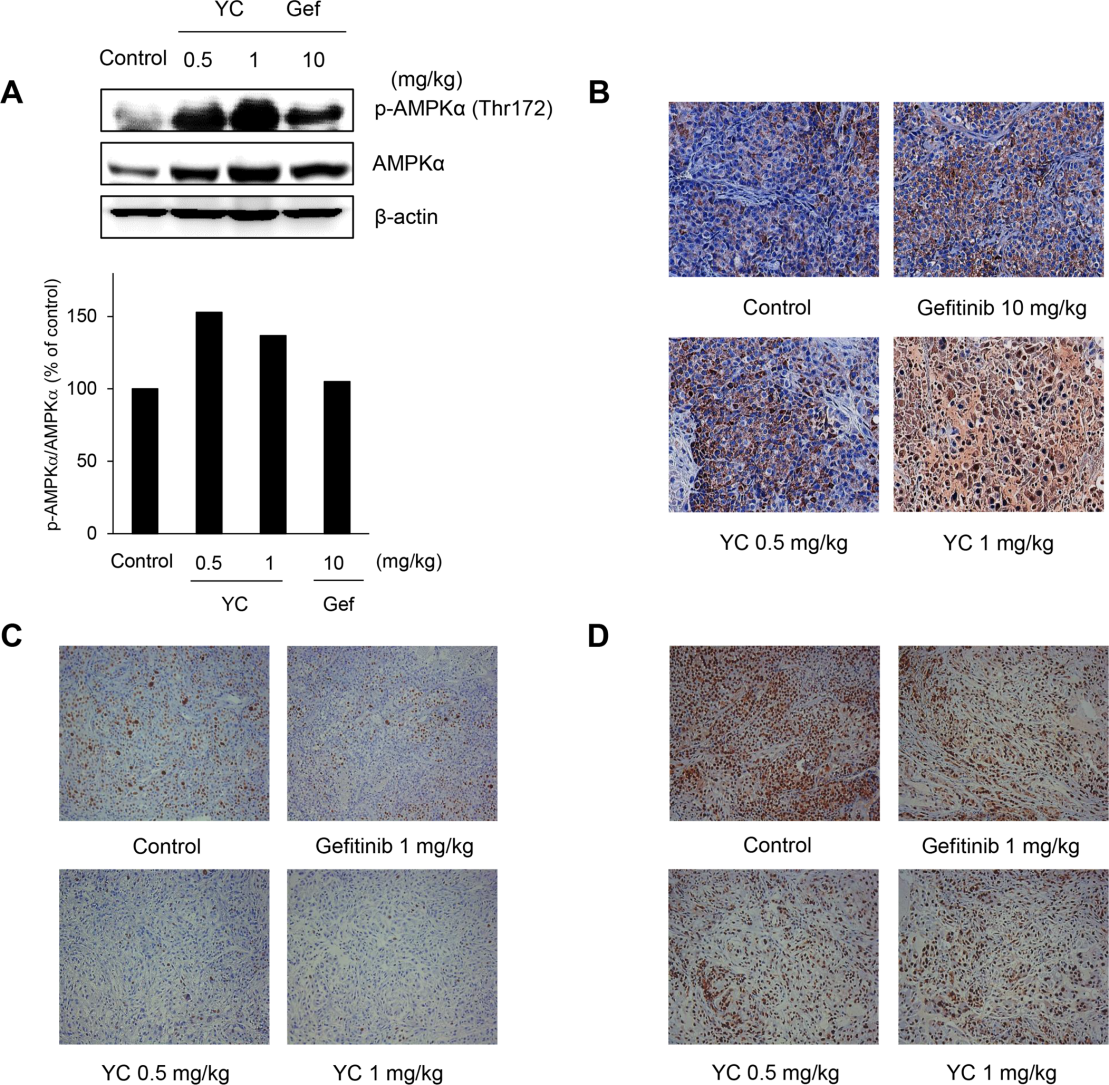
Effects of YC on the expression of AMPK and proliferative biomarkers in H1993 xenograft tumors. The protein extracts were derived from tumor tissues of H1993 xenograft model. (A) The AMPK expressions were measured by Western blot analysis. (B) The p-AMPK expression was measured by immunohistochemistry. (C) The Ki-67 expression was measured by immunohistochemistry. (D) The PCNA expression was measured by immunohistochemistry.

In addition, a biochemical analysis of the tumor tissues also confirmed the activation of AMPK by the treatment of YC using Western blot (Fig 7A) and immunohistochemical analysis (Fig 7B) in a dose-dependent manner. YC also exhibited the inhibition of the expression of the proliferation biomarkers Ki-67 (Fig 7C) and proliferating cell nuclear antigen (PCNA) (Fig 7D) in tumor tissues. These data suggested that YC effectively suppressed the tumor growth of the H1993 cells *in vivo*, and its anti-tumor activity might in part be associated with the activation of AMPK signaling pathway.

## Discussion

Natural products have been played an important role in drug discovery and development [21]. In particular, plant-based terrestrial natural products have been valuable sources of therapeutic agents. Yuanhuacine (YC), a daphnane diterpenoid, is a principle component of the flowers of *Daphne genkwa*. Recent studies by our group and others reported the strong anti-proliferative effects of YC against various cancer cell lines [8, 22–24]. One plausible mechanism includes the targeting of topoisomerase-DNA complexes, and YC is considered as a DNA-damaging agent [25]. YC also showed the induction of G2/M phase cell cycle arrest in human bladder and colon cancer cells [26]. However, the precise molecular mechanism of YC in the anticancer activity of human lung cancer cells, especially, NSCLC cell growth remained to be elucidated. This study demonstrates that YC regulates the AMPK/mTOR signaling pathway and inhibits actin cytoskeleton organization in human lung H1993 cancer cells.

Recent studies revealed that AMPK/mTOR signaling pathways are strongly connected in the regulation of cancer cell growth, proliferation, and survival. In particular, the activation of AMPK negatively regulates the cancer cell growth through downstream mTOR signaling pathways [26]. Indeed, many natural products-derived compounds including galegine from *Galega officinalis* [27], resveratrol from red grapes, quercetin present in many fruits and vegetables, ginsenoside from *Panax ginseng*, curcumin from *Curcuma longa*, berberine from *Coptis chinensis*, epigallocatechin gallate from green tea, theaflavin from black tea [28], and hispidulin from snow lotus [29] have exhibited the anticancer activity by the activation of AMPK pathways. In the present study, we also found that YC effectively activates the AMPK and downregulates the mTORC2-associated signaling pathways. The activation of AMPK by YC was found to be occurred in an early event in the H1993 cells and the activation of AMPK was also confirmed by the co-treatment with an AMPK inhibitor (compound C) or an activator (metformin). These findings suggest that the anti-proliferative activity of YC is in part associated with the activation of AMPK in the human NSCLC cells.

AMPK displays many downstream target such as ACC, mTOR, p-p53, and COX-2 in cancer cells. Among them, ACC is a well-established downstream substrate of AMPK involved in lipid metabolism [30]. In general, the activity of ACC is known to be negatively regulated by AMPK [31]. However, several studies have reported that ACC is a common characteristic of cancers and the specific knock down of ACC inhibits cell proliferation and induces apoptosis in cancer cells [32–34]. In the present study, the level of p-ACC was significantly decreased by YC in a concentration-dependent manner, suggesting that YC suppresses p-ACC via the AMPK-independent pathway and dephosphorylation of ACC might be able to affect the growth of cancer cells. However, the detailed mechanisms of the downregulation of p-ACC by YC remains to be investigated.

Tuberous sclerosis complex 2 (TSC2) tumor suppressor and its obligate partner TSC1 are reported as upstream components of mTORC1 [35]. AMPK directly phosphorylates TSC2 when the levels of ATP, glucose or oxygen are low [26, 36, 37]. Recent findings also revealed that TSC1-TSC2 complex promotes mTORC2 activity and this event is occurred independent of its inhibitory effects on mTORC1 [38, 39]. mTORC2 phosphorylates Akt at Ser473 [40], and phosphorylation of Akt negatively regulates the function of TSC1-TSC2 [41]. In the present study, we found that AMPK was up-regulated, and mTORC2-associated Akt activation was inhibited by YC. These data indicate that YC may induce the negative feedback loop of TSC1-TSC2, mTORC2 and Akt in the H1993 cells. Further studies are warranted to address how YC induces AMPK and TSC1-TSC2 complex that may be involved in the inhibition of the mTORC2-Akt pathway.

mTOR, mammalian lethal with SEC13 protein 8 (mLST8), and DEP domain-containing mTOR-interacting protein (Deptor) are components of both mTORC1 and mTORC2. mTORC1 and mTORC2 are defined by the binding of raptor and rictor to the mTOR protein, respectively. mTORC2 also contains the additional proteins including mammalian SAPK interacting protein (mSIN1) and Protor [42]. The phosphorylation of rictor at Thr1135, one of the mTORC2 components, inhibits mTORC2-dependent phosphorylation of Akt [43]. However, YC did not affect the phosphorylation of rictor at Thr1135 and phosphorylated rictor at Ser1219 (Fig 3B). Although the role of phosphorylation at Ser1219 site remains poorly characterized, the enhanced phosphorylation of rictor at Ser1219 could affect mTORC2. In addition, YC induced the disruption of rictor-mTOR association (Fig 3C). This disruption of the complex occurred the inhibition of mTORC2 signaling pathway including PKC-α, Rac1, and F-actin. These proteins are also strongly related with the regulation of actin cytoskeleton organization that is critical in migration, invasion, survival, colonization, adhesion, and metastasis in cancer cells. These effects might be associated with the inhibition of cell migration and invasion by YC in the H1993 cells.

Epithelial-mesenchymal transition (EMT) is a primary step to induce tumor cell invasion and metastasis [44–46]. E-cadherin is a hemophilic cell-to-cell adhesion protein localized to the adherent junctions of all epithelial cells, and is down-regulated when EMT is induced [47, 48]. In addition, EMT is associated with actin cytoskeleton [49, 50]. When examined the expression of E-cadherin in the H1993 cells YC significantly increased the induction of E-cadherin, suggesting the possibility of EMT induction and thus inhibition of cell migration and invasion of cancer cells.

In addition, anti-tumor activity of YC was also confirmed in the nude mouse xenograft model bearing the H1993 cells. The analysis of tumor tissues indicated that YC effectively up-regulated the AMPK activation in *in vivo* tumor tissues. This is well correlated with the findings *in vitro* cell culture systems. These data suggest that anti-proliferative effects of YC in NSCLC cells might be in part highly correlated with the up-regulation of AMPK signaling pathways.

In summary, we herein demonstrate that the potential anti-proliferative activity of yuanhuacine against human NSCLC cells might be in part associated with the modulation of the AMPK/mTORC2 signaling pathways. Therefore, YC is considered to be prioritized in the development of anticancer agents derived from natural products.
